# Breaking Barriers: Exploiting Envelope Biogenesis and Stress Responses to Develop Novel Antimicrobial Strategies in Gram-Negative Bacteria

**DOI:** 10.3390/pathogens13100889

**Published:** 2024-10-11

**Authors:** Renu Bisht, Pierre D. Charlesworth, Paola Sperandeo, Alessandra Polissi

**Affiliations:** Department of Pharmacological and Biomolecular Sciences, University of Milano, 20133 Milano, Italy; renu.bisht@unimi.it (R.B.); pierre.charlesworth@unimi.it (P.D.C.); alessandra.polissi@unimi.it (A.P.)

**Keywords:** envelope stress response, two-component system, outer membrane biogenesis, cell envelope assembly, antimicrobial resistance, lipopolysaccharide, outer membrane proteins, TCS inhibitors, Gram-negative bacteria

## Abstract

Antimicrobial resistance (AMR) has emerged as a global health threat, necessitating immediate actions to develop novel antimicrobial strategies and enforce strong stewardship of existing antibiotics to manage the emergence of drug-resistant strains. This issue is particularly concerning when it comes to Gram-negative bacteria, which possess an almost impenetrable outer membrane (OM) that acts as a formidable barrier to existing antimicrobial compounds. This OM is an asymmetric structure, composed of various components that confer stability, fluidity, and integrity to the bacterial cell. The maintenance and restoration of membrane integrity are regulated by envelope stress response systems (ESRs), which monitor its assembly and detect damages caused by external insults. Bacterial communities encounter a wide range of environmental niches to which they must respond and adapt for survival, sustenance, and virulence. ESRs play crucial roles in coordinating the expression of virulence factors, adaptive physiological behaviors, and antibiotic resistance determinants. Given their role in regulating bacterial cell physiology and maintaining membrane homeostasis, ESRs present promising targets for drug development. Considering numerous studies highlighting the involvement of ESRs in virulence, antibiotic resistance, and alternative resistance mechanisms in pathogens, this review aims to present these systems as potential drug targets, thereby encouraging further research in this direction.

## 1. Introduction

Antimicrobial resistance (AMR) poses a critical threat to human health, economies, and food security globally. In 2019 alone, over 1.25 million deaths were linked to bacterial AMR, with projections indicating a potential rise to 10 million by 2050 without swift intervention [1]. This crisis leads to increased hospitalizations, financial burden, and a concerning regression towards a pre-antibiotic era. To explain how we have reached this state, it is imperative to understand the mechanisms of AMR, the driving forces, and current state of new antimicrobial drug development. AMR is a natural evolutionary process which selects for the most adapted strains in response to antimicrobial selection pressure, following which the selected strains can better resist the antimicrobials compared to their predecessors and transfer the resistance determinants to successive generations. However, the rate of AMR has surged in recent times, majorly due to man-made factors such as inappropriate usage of antibiotics in humans and animals, climate change, and pollution from pharmaceuticals [2]. These forces have contributed to the rise of the most notorious antimicrobial resistant ESKAPE pathogens (*Enterococcus faecium*, *Staphylococcus aureus*, *Klebsiella pneumoniae*, *Acinetobacter baumannii*, *Pseudomonas aeruginosa*, and *Enterobacter* species), which call for urgent development of novel antibiotics [3].

Gram-negative bacteria (GNB), in particular, are major contributors of antibiotic-resistant infections and infection-associated deaths, due to their intrinsic resistance to many antibiotics, facilitated by a unique cell envelope that makes them impermeable to these molecules. The homeostasis of the complex envelope of GNB is maintained by signal transduction pathways known as envelope stress responses (ESRs). ESRs are crucial for ensuring the fidelity of envelope assembly during normal physiological growth and orchestrating specific responses to detect and repair envelope damage resulting from external insults, such as protein toxins, immune factors, and antimicrobial compounds [4].

In addition to intrinsic resistance, there are four main recognized modes of AMR acquisition in bacteria: (1) mutations altering the target of the antimicrobial drug; (2) gene expression changes in genes encoding efflux pumps; (3) acquisition of new genetic information responsible for expression of genes encoding enzymes for drug modification; and (4) specific membrane remodeling to hinder drug influx at the cell surface [5].

This review summarizes our knowledge of the biogenesis of the outer membrane in GNB responsible for intrinsic antimicrobial resistance and focuses on the fourth mode of AMR acquisition in GNB, exploring surveillance and maintenance processes of cell envelope integrity. Finally, we will discuss how these pathways could be leveraged to restore bacterial sensitivity to existing drugs.

## 2. Envelope of Gram-Negative Bacteria

Under an electron microscope, the *Escherichia coli* cell envelope appears as a double-layered structure: the inner layer consists of a canonical fluid-mosaic cytoplasmic or inner membrane (IM), while the outer layer is the asymmetric OM adorned with lipopolysaccharide (LPS) on the outer leaflet. The cytoplasmic IM is a phospholipid bilayer containing mostly apolar α-helical proteins that are involved in a diverse array of functions, from ATP hydrolysis and lipid biosynthesis to nutrient uptake and efflux. The IM also facilitates the provision of all OM envelope and periplasmic components through either direct synthesis within the membrane, or transport from the cytoplasm across the IM via translocation, secretion, or flippases [6]. The aqueous periplasmic space between the IM and the OM is an oxidative environment that hosts cysteine-rich proteins with a multitude of roles, such as protein folding, and enzymatic sugar and amino acid transport [7]. The periplasm also harbors the peptidoglycan (PG) layer, a rigid mesh-like network of glycan strands cross-linked by short peptides. This matrix plays an important role in withstanding turgor pressure, as well as in maintaining the defined cell shape and the scaffolding for other cell envelope elements [8]. In GNB, the PG layer is comparatively thinner than that of Gram-positive bacteria and it is covalently linked to the OM [9].

In contrast to the IM, the OM is an asymmetric membrane comprising an inner leaflet of phospholipids and an outer leaflet that is mostly populated with LPS. Comprising a 4–7 acyl chain lipid A, a variable length core oligosaccharide, and a distal repeating chain polysaccharide called the O-antigen, LPS molecules are tightly packed in the outer leaflet, making the GNB cell envelope impermeable to polar and non-polar compounds [10]. Notably, in some bacterial species such as *A. baumannii* the O-antigen is not present, which results in the production of molecules with only lipid A and the core (known as lipooligosaccharides or LOSs) [11]. The LPS molecules are tightly associated with one another due to divalent Mg^2+^ and Ca^2+^ cations which link the negative charges of lipid A and core moieties of nearby LPS molecules [12,13]. This tightly packed arrangement of the LPS molecules, attributed to the strong electronic interaction and saturation of fatty acid chains, provides additional rigidity to the OM which contributes to withstand the turgor pressor with PG [14]. Currently, cationic polymyxins are used as last resort antibiotics in a clinical setting against GNB, as they bind to LPS and disrupt the interaction between them, resulting in permeabilization of the membrane [15].

The asymmetry of the OM is ensured by the active transport of LPS from the site of synthesis (the cytoplasm and IM) to the outer leaflet of the OM by the Lpt system (discussed in Section 2.1.2), while the maintenance of lipid asymmetry (Mla) system is responsible for the retrograde transport of PLs across the two membranes and for counteracting PLs flipping from the inner to the outer leaflet of the OM (discussed in Section 2.1) [16]. Additionally, OM phospholipase A (PldA) acts as a sensor for OM lipid asymmetry by degrading mislocalized PLs and generating a signal to increase LPS production in response [17]. Flux of molecules through the OM is mediated by OM proteins (OMPs), most of which are cylindrical β-barrels. The functionality of these OM transmembrane proteins varies from selectively transporting small cationic and anionic molecules (OmpF/OmpC and PhoE, respectively) to localization of the OM components themselves (e.g., at the endpoints of the OM envelope assembly complexes). Lipoproteins anchored to the OM by a lipid moiety are also present in the OM [18]. OM envelope components are assembled through tightly regulated biogenesis and localization pathways which are interdependent to ensure coordinated OM growth and can be dynamically modulated when the cell is under stress [19].

### 2.1. Envelope Assembly Pathways for Outer Membrane Biogenesis

**Figure 1:**
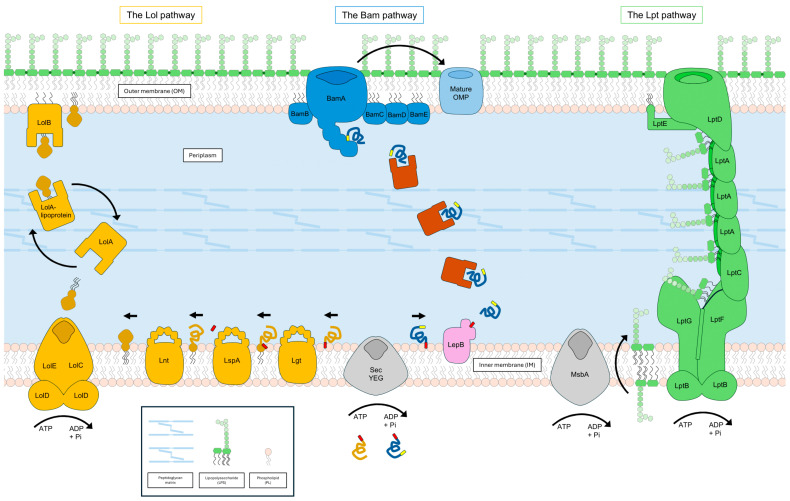
Summary of outer membrane biogenesis pathways in Gram-negative bacteria. The following elements are represented in different colors: the Lol pathway in yellow, the Bam pathway in blue, and the Lpt pathway in green. The gray IM complexes SecYEG and MsbA are not directly members of these pathways but contribute through translocation of their substrates. Unfolded proteins destined for the Lol and Bam pathway are shown below SecYEG in yellow and blue, respectively. The signal sequences are shown at the N-terminus in red, and the β-signal sequence is shown in the unfolded Bam protein in yellow. The pink molecule LepB located in the IM is the signal transpeptidase that catalyzes the removal of the signal sequence from unfolded outer membrane proteins. The molecules shown in orange represent the various outer membrane protein chaperones. The box in the bottom left of the figure depicts the legend representing LPS, PL molecules, and the peptidoglycan matrix layer.

The biosynthesis of OM components begins in the cytoplasm (synthesis of protein and LPS precursors), where there is availability of building blocks and energy; from there, dedicated machineries allow the transport of these components across the oxidative periplasm and enable their assembly into the asymmetric bilayer of the OM. The three most well-known OM assembly systems that ensure this transport are the Bam, Lpt, and Lol systems (which transport OMPs, LPS, and lipoproteins, respectively, and the mechanisms of which will be discussed in further detail in the following sections; see Figure 1). Some of these systems are intricately connected at the metabolic precursor level, sharing metabolic intermediates (e.g., LPSs and phospholipids (PLs) share a common precursor substrate [20]; phospholipids can serve as donors for acylation in lipoprotein maturation [21]). Additionally, some components of one transport system are assembled and/or transported by other transport systems; for example, Bam assembles the OMP LptD involved in LPS assembly whereas Lol transports lipoproteins such as LptE and BamD [18,22]. This degree of interconnectedness invariably causes a cascade of effects across multiple pathways in the event of cell envelope damage or regulatory modulation.

The maintenance of both OM lipid asymmetry and continued OM growth requires specific transport systems to ensure that PLs are delivered and dynamically removed, as and when needed. For example, PLs can be mislocalized to the outer leaflet of the OM which can increase permeability and compromise the integrity of the OM. Thus, both anterograde (for growth) and retrograde (to remove mislocalized PLs) transport systems are needed to preserve lipid asymmetry. Anterograde transport of PLs is achieved through a number of protein complexes, including AsmA-like proteins which contain a hydrophobic groove (similar to that of LptA, discussed in more detail in Section 2.1.2) that shields the lipid moiety of PL during periplasmic traversal [23,24]. Additionally, a polypeptide MCE domain transporter known as LetB has recently been identified that forms a dynamic hydrophobic tunnel large enough to allow PL transport [25]. Retrograde transport is thought to primarily be achieved by the Mla pathway, consisting of ABC transporter MlaFEDB, periplasmic lipid chaperone MlaC, and OM complex MlaA-OmpC/F [26]. Readers interested in PL transport can refer further to these comprehensive articles [27,28].

Different OM assembly systems are preferentially regulated by specific ESRs (σ^E^, Rcs, and Cpx) that dynamically monitor the transport and assembly of the OM components and control the expression of related genes during environmental or intrinsic stresses. For example, the σ^E^ system is activated in response to disruption of OMP biogenesis, through proteolysis of the anti-σ factor RseA upon sensing the unfolded OMPs (uOMPs) accumulated in the periplasm [29]. Intact RseA tethers the alternative RNA polymerase σ^E^ factor to the membrane and its proteolysis results in the release of σ^E^ into the cytoplasm. Consequently, the activation of σ^E^ modulates the transcription of a variety of genes, leading to decreased OMP expression, improved transport and assembly of OMPs and, the degradation of periplasmic uOMPs [29]. Similarly, a perturbation in LPS molecular arrangement by cationic antimicrobial peptides (e.g., polymyxin B) can induce the Rcs system to promote colanic acid production to help stabilize the LPS layer [30]. In another example, when the OM-anchored lipoprotein NlpE is mislocalized, its resulting IM accumulation activates the Cpx response pathway which provides a protective effect against lipoprotein trafficking perturbation [31]. In this way, ESRs can ameliorate many of the effects of antibiotics that target OM biogenesis and are frequently implicated in mechanisms of antimicrobial resistance.

#### 2.1.1. OMP Transport via Bam

Many OMPs are essential to GNB, and their misfolding or mislocalization can be detrimental to the cell. In fact, as already mentioned, two of the OM assembly systems (Bam and Lpt) feature OMPs that must be properly inserted into the OM in order for the OM itself to be maintained (Figure 1). Consequently, interrupting the folding and transport of OMPs represents a desirable objective in the context of disrupting OM biogenesis. Newly synthesized uOMPs must first pass through the cytoplasm to the periplasm. The N-terminal of nascent uOMPs contains a signal sequence that is recognized by the SecYEG translocon, which transports them either during translation or post-translationally across the IM and into the periplasm [32]. Once there, the signal peptide tethers the uOMP to the IM, which must then be cleaved by signal peptidase I (in *E. coli*, LepB) so that the unfolded protein is transported to the OM. The uOMPs remain in their unfolded state until they reach the OM, but accumulation of uOMPs in the periplasm is toxic, triggering the σ^E^ stress response. In order to mitigate the toxic build-up of uOMPs in the periplasm, several chaperones present in the periplasm bind to and transport these uOMPs to the OM-bound Bam complex. Of these periplasmic chaperones, most OMPs seem to prefer SurA, which plays a vital role in virulence of Gram-negative pathogenic bacteria, although other chaperones perform more specific roles in OM biogenesis (for example, the role of Skp in LptD assembly) [33,34,35,36].

Next, the periplasmic chaperones finally deliver the uOMPs to their destination at the Bam complex, which mediates their folding into the β-barrel configuration and assists in their insertion into the OM. The Bam complex comprises five proteins (BamA-E), of which BamA is itself a β-barrel OMP. BamB-E are all lipoproteins that are scaffolded by the POTRA (polypeptide transport-associated) domains of BamA [37]. BamA and BamD are essential components of the Bam machinery and appear to be well-conserved across many different bacterial species, whereas BamB, BamC, and BamE are non-essential [38]. BamD accepts uOMPs from the periplasmic chaperones by recognizing the β-signal peptide at their C-terminus and, together with BamA, folds the uOMPs into β-barrels [39]. BamA features structural elements that allow it to adopt two conformations: either an outward-open gate conformation or an inward periplasmic-open conformation [40]. This conformational functionality allows the Bam complex to deliver folded OMPs to the outer leaflet of the OM whilst simultaneously preventing the entry of harmful molecules to the cell. Recently, BamA has been shown to interact with and assist in the transport of RscF (a sensor lipoprotein part of the Rcs ESR)–OMP complexes to the cell surface, raising the question of whether other outer membrane lipoproteins are also transported through the Bam machinery [41].

#### 2.1.2. LPS Transport via Lpt

As mentioned previously in Section 2, amphipathic LPS is a vitally important OM structural component for most GNB, providing both structural support and membrane impermeability. However, the fatty acyl chains within the lipid A moiety of LPS pose a challenge: the LPS molecules must traverse from the cytoplasmic site of biosynthesis to their destination at the OM. In order to cross the aqueous periplasm between the IM and OM, the hydrophobic lipid A moiety must be sheltered from the hydrophilic surroundings and cannot be transported unassisted [42]. This shielded transport is mediated by the Lpt machinery, a system comprised of seven essential proteins that form a complex across the IM (LptB_2_FGC), periplasm (LptA), and OM (LptDE) [43] (Figure 1). The lipid A core of LPS is synthesized at the cytoplasmic leaflet of the IM and is flipped to the periplasmic leaflet of the IM via the ABC-transporter MsbA [44]. If present, the O-antigen is synthesized independently within the cytoplasm, transported to the periplasm, and then attached to the lipid A core via the ligase WaaL, resulting in full-length LPS [45]. In the IM, the ABC transporter LptB_2_FGC is composed by two nucleotide binding domains (LptB_2_) which bind and hydrolyze ATP, two transmembrane subunits (LptF and LptG) which form the LPS binding cavity, and the unconventional regulatory subunit LptC [46]. Entry of LPS from the periplasmic leaflet of the IM into the Lpt machinery occurs in an energy-independent fashion, then the homodimeric LptB binds ATP and induces a conformational change. This movement transmits subsequent conformational changes to both transmembrane proteins, LptF and LptG, which are each coupled to a single monomer of LptB_2_. Thus, binding to ATP induces the extraction of LPS from the IM and its funneling into the periplasmic cavity of LptF. Hydrolysis of the bound ATP triggers the LptB_2_FG subcomplex to return to its resting state, closing the cavity and extruding the LPS out of the IM and into LptC [47]. The LptC protein is connected directly with the periplasmic cavity of LptF, forming a continuous channel, along which the fatty acyl chains are shielded throughout their passage towards the periplasm. LptA acts as a periplasmic bridge, connecting with LptC and spanning the periplasm to allow movement of LPS through the aqueous space [48]. Multiple monomers of LptA are able to oligomerize to form the periplasmic bridge, but the exact number is unknown in vivo [49]. The OM subcomplex of Lpt comprises LptD (a β-barrel protein that forms a channel through the OM) and LptE (a lipoprotein embedded within the lumen of LptD that acts as a “plug”) [50]. The periplasmic domain of LptD receives LPS from the C-terminus of LptA, and directly inserts the lipid moiety of LPS into the OM through an opening between the periplasmic domain and the β-barrel domain [51]. The mechanism of the Lpt machinery has been compared to that of a PEZ candy dispenser, in which the candies represent the LPS molecules and the Lpt complex is likened to the spring-loaded candy dispenser. Once a stack of PEZ candies is inserted into the central cavity, the spring-loaded platform at the base of the stack ensures that a new candy is pushed to the top whenever a candy is consumed. In the analogous Lpt system, the spring-loaded platform is comparable to the extrusion pressure produced by the LptB_2_FG subcomplex, continually pushing new LPS molecules into the LptCAD channel and ultimately through the lumen of LptD and out to the OM—hence, the “PEZ model” of LPS transport via Lpt [43].

#### 2.1.3. Lipoprotein Transport via Lol

Over a third of the proteins produced in the GNB model organism *E. coli* are destined for the cell envelope; many of these are either soluble proteins that perform their functions within the periplasm or they are membrane-integral proteins such as the OMPs discussed in Section 2.1.1 [52]. Additionally, there is a collection of proteins that are anchored to a membrane by a lipid moiety known as lipoproteins [37]. Lipoproteins perform many important functions, such as OM integrity sensing (e.g., RscF and NlpE), performing critical steps within OM biogenesis complexes (e.g., BamD and LptE), and PG synthesis and remodeling (e.g., LpoA and LpoB) [53,54]. Most of these OM lipoproteins are transported from the IM by the Lol system (Figure 1). As with most membrane proteins in GNB, lipoprotein precursor synthesis is carried out in the cytoplasm. Lipoprotein precursors are characterized by the presence of a signal sequence that contains a lipobox, comprising a conserved cysteine that is acylated in the mature form [55]. Most of these unfolded lipoprotein precursors travel to the IM via the general secretory system, or Sec [56]. Following the translocation of lipoprotein precursors across the IM, lipoprotein maturation is mediated by three IM proteins (Lgt, LspA and Lnt) that catalyze three distinct maturation reactions that include lipid modifications and cleavage of the signal peptide [57]. Mature lipoproteins are then sorted into groups destined for the IM or OM through a sorting signal localized at the +2 position amino acid, relative to the N-terminal Cys residue. Briefly, if the residue at this position is Asp, the lipoprotein will be localized to the IM, but replacement of this residue with any other amino acid results in localization to the OM [58]. Additionally, the residue at the +3 position can have a further influence on the localization of the lipoprotein, causing localization to the IM with some combinations of residues at +2 and +3 even in the absence of Asp [59]. This system of lipoprotein sorting is known as the “+2 rule” and is well conserved in Enterobacteriaceae [60].

If a lipoprotein is sorted for transport to the OM, the Lol pathway (composed by LolA-E proteins) is recruited to enact the translocation. The first step in the process is extraction of the lipoprotein from the IM by the ABC transporter LolCDE complex in an ATP-dependent manner, and the next is the release of the lipoprotein to the periplasmic LolA to form a water-soluble stoichiometric complex [61]. In a similar manner to the LPS molecule discussed previously, the acyl chains of the lipoprotein must be protected during transport across the aqueous periplasm; this protection is provided by a large hydrophobic cavity within LolA [62]. Once the soluble LolA–lipoprotein complex has traversed the periplasm, it delivers the lipoprotein to the OM bound acceptor protein LolB [63]. LolB has a similar structure to LolA, with a hydrophobic cavity that can accommodate the acyl chains of lipoprotein, but with the important difference that the affinity between LolB and lipoprotein is higher [64]. Due to these affinity differences between LolA and LolB, an affinity-based handover of lipoprotein can occur from LolA to LolB, which causes LolA to return to a closed conformation and return to the LolCDE complex to accept a new lipoprotein. LolB then completes the transport of lipoprotein by anchoring it to the OM [65].

### 2.2. Envelope Assembly Pathways as Antibiotic Targets in Gram-Negative Bacteria

Given that most of the OM assembly pathways described are essential in many GNB, it is reasonable to consider that directly inhibiting these pathways might be a compelling strategy for developing antibiotic compounds. In this section, we highlight several examples of such approaches that exploit these pathways.

The folding and localization of OMPs via Bam has been targeted using a few different methods. Directly targeting and inhibiting BamA has been attempted by several research groups; through several distinct mechanisms, including binding the lateral gate in BamA and disrupting the BamA-BamD interaction, therefore preventing the folding of OMPs [66,67,68]. Cytoplasmic expression of a β-signal peptide derived from BamA that binds BamD and inhibits its activity has been shown to disrupt the Bam machinery in vivo, consequently reducing OMP transport and permeabilizing the OM. This rationale could be utilized to develop an antibiotic via synthetic mimicry of β-signal peptides that target BamD activity [69]. In addition to targeting Bam itself, members of the chaperone network could also be a favorable target. Recently, the key role of periplasmic chaperone SurA assisting Bam in the delivery of OMPs has been shown to cause OM assembly defects when disrupted [70]. A study by Bell et al. has revealed a promising inhibitor of the periplasmic chaperone SurA through in silico screening, which targets a binding site that inhibits its activity and thus interferes with the folding of OMPs [71].

The LPS transport pathway can be targeted at various stages as part of an antibiotic drug discovery approach [72]. Targeting the translocation of LPS from the IM to OM via MsbA has been found to be a feasible strategy. The compound G907, which inhibits the function of MsbA by “wedging” into a transmembrane pocket and trapping it in a cytoplasmic-facing conformation, exemplifies this approach [73]. Member proteins of the Lpt complex can also be targeted as a viable therapeutic approach to disrupt LPS biogenesis. Thanatin, an antimicrobial peptide originally derived from insects, has been shown to disrupt the LptCAD complex by binding to the interaction sites, inhibiting complex formation and thereby halting LPS transport [74,75]. Similarly, a macrocyclic peptide known as Zosurabalpin has been identified that traps LPS within the LptB_2_FGC complex and disrupts LPS transport. Although the study was conducted in *A. baumannii* (which does not require LPS for viability), the concept could be developed to inhibit LPS transport in other LPS-dependent GNB strains [76,77]. Interestingly, in *A. baumannii*, it is the accumulation of LPS in the IM, rather than the blocking of LPS transport to the OM, that presumably exerts the killing effect on the cells. Accordingly, inactivation of the IM LPS flippase MsbA is toxic in this organism due to LPS mislocalization in the IM [78]. Another macrocyclic peptide called murepavadin, specifically targeting *P. aeruginosa* LptD to disrupt LPS transport, is currently in its second phase 3 clinical trial as an inhaled formulation which was unsuccessful in the first phase 3 trial due to increased nephrotoxicity upon intravenous administration [79]. Combinatorial drug therapies also hold potential for overcoming antibiotic resistance. For example, it has been shown that the aminocoumarin antibiotic novobiocin (primarily bactericidal due to its action against DNA gyrase) also stimulates the ATP hydrolysis of LptB. Stimulation of LptB alone is non-toxic to GNB, but novobiocin synergistically enhances the efficacy of polymyxin treatment, potentially leading to reduced dosages, possible resistant strain re-sensitization, and decreased nephrotoxicity in clinical settings [80].

Similarly, the Lol pathway, responsible for lipoprotein transport, is another attractive target. LolCDE is relatively well conserved and essential amongst GNB, and several compounds have been developed in the last decade via screening strategies that inhibit LolCDE, leading to toxic accumulation of lipoproteins at the IM [81,82,83]. Recently, Buss and colleagues identified a molecule called MAC13243 (and its A22-like degradation product) during a screen for inhibitors of the PG elongasome protein MreB [84]. The MAC13243 molecule was initially suggested to have activity against LolA as overexpression of the *lolA* gene suppressed its lethal effect [85]. However, it was found that the suppression was abrogated in Δ*rscF* mutants, suggesting that it was in fact the induction of the Rcs response via *lolA* overexpression that was the causal factor of MC13243-mediated suppression (see Section 2.3.1 for Rcs description) [84,86]. A brief overview of inhibitor compounds that target OM biogenesis pathways can be found in Table 1.

Though OM assembly machineries and their components offer exciting opportunities to develop novel antibiotic strategies, more robust approaches are needed to circumvent the issues of antibiotic resistance and toxicity concerns in the host. The OM biogenesis pathways described in the previous sections are closely monitored and regulated by ESRs, which are able to sense changes in OM integrity (either through environmental stress, protein misfolding, or disruption in the OM biogenesis pathways) [19]. Once a change in OM integrity or any perturbation in the cell envelope is sensed, these signaling networks are activated in response to restore homeostasis and allow the GNB to adapt to hostile environments, including the presence of antibiotics. It is this activity of ESRs and their fine interplay with OM biogenesis pathways that makes them attractive targets for therapeutic intervention aimed at preventing the adaptation of bacterial cells to antimicrobial pressure, thus enhancing the efficacy of existing antibiotics. In the following section of this review, we will explore some of the ESR pathways and their corresponding signal transduction systems in the context of antibiotic resistance, as well as how these systems can be exploited to develop antimicrobial strategies.

### 2.3. ESR Pathways as Major Determinants of Cell Envelope Integrity, Virulence, and Pathogenesis in Gram-Negative Bacteria

Cell envelope stress can arise due to either environmental changes, such as increased osmolarity, redox stress, and exposure to harmful chemical compounds, antimicrobials, or to intrinsic stress factors, such as defects in the biosynthesis and transport of cell envelope components, protein misfolding, and mutations [88]. ESRs are the signal transduction pathways deputed to monitor and respond to the changing environment counteracting various stresses. Based on their protein composition and mechanism of signal transduction, the ESRs can be grouped into two component signal transduction systems (TCSs) and RNA polymerase-associating alternative sigma factors. The best-studied envelope stress-related sigma factor in *E. coli* is σ^E^, whose regulation and mechanism has been described in Section 2.1. TCSs instead are typically comprised of a sensor histidine kinase (HK) localized at the IM that senses the environmental input signals, and a cognate response regulator (RR) that responds to the input signal by regulating the expression of genes (output signal) related to adaptation and resistance [89]. These pathways aid GNB cells in maintaining various physiological states such as adhesion, virulence, microbial warfare, and biofilm formation in order to respond and adapt to different environments, including the host [90]. In the presence of harmful agents, such as effector molecules of the immune system (cationic antimicrobial peptides and lysozyme) or antibiotics, the GNB cell reacts by inducing an intricate and complex response involving the fine tuning of several ESRs, namely σ^E^ and the TCSs Cpx, Rcs, Bae, and Pho, to name a few [4]. Different ESRs are activated by specific perturbations in different OM components. The CpxR and Rcs pathways have been found to be two major ESRs that are triggered by inhibitors targeting PG (such as β-lactams and A22) and are also involved in the generation of multidrug resistance (MDR) phenotypes [19]. The MDR emergence is majorly due to the Cpx-mediated activation of MDR efflux pumps [91,92,93]. The Cpx pathway also monitors lipoprotein biogenesis through the OM sensory lipoprotein NlpE, which upon mislocalization to the IM triggers and activates the Cpx regulon. This activation has been shown to exert a protective effect against the lipoprotein biogenesis inhibitors [94]. On the contrary, under conditions of lipoprotein biogenesis inhibition*,* the mislocalization of RcsF—an OM sensory lipoprotein of the Rcs system—affects cell survival as a result of the induction of the Rcs system and the consequent accumulation of higher levels of Rcs-induced OsmB lipoprotein in the IM [88].

OMP biogenesis is primarily regulated by σ^E^, Cpx, and Rcs, where σ^E^ is the main ESR controlling the activity of OMP biogenesis pathways. OMP assembly defects caused by Bam impairment result in the accumulation of unfolded OMPs in the periplasm. As previously mentioned, unfolded OMPs induce σ^E^-mediated expression of several genes implicated in the adaptive cellular response designed to address the OMP assembly defects through various mechanisms, including downregulation of new OMPs expression, assisted folding of the unfolded OMPs present in the periplasm and insertion into the OM, and degradation of the unfolded OMPs [88]. LPS defects induce primarily Rcs and act synergistically with protein misfolding to activate σ^E^ [95].

Other ESRs are induced by generic stresses that impact the envelope and help maintain OM homeostasis. For example, the Bae system is activated in response to toxic molecules such as ethanol, nickel chloride, zinc, and sodium tungstate, and its activation leads to upregulation of genes encoding periplasmic chaperones and efflux pumps [88]; meanwhile, the Psp system responds to stressors such as infection by filamentous phages, extreme heat shock, osmotic shock, organic solvent exposure, disruption of protein secretion, and localization of OMPs at the IM, which leads to the transcription of the *psp* genes to counteract these stresses [96].

**Figure 2:**
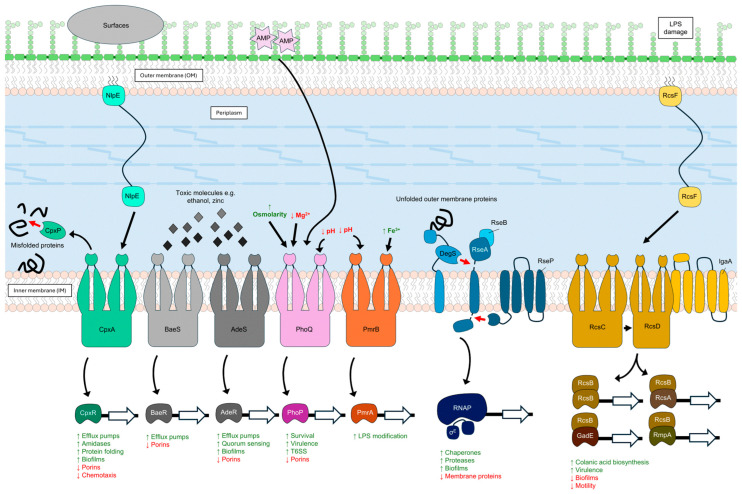
Summary of envelope stress response pathways and two component systems in Gram-negative bacteria. CpxAR is shown in green, BaeSR in light gray, AdeSR in dark gray, PhoPQ in pink, PmrAB in orange, σ^E^ in blue, and Rcs in yellow. Below each system is an overview of the processes regulated following activation of the envelope stress response: green represents upregulated processes and red represents downregulated processes. The molecular framework of the cell envelope remains consistent with the legend depicted in the bottom left box in Figure 1.

Remarkably, each ESR perceives specific changes in the cell envelope and mediates the protective, reparative, and defensive response against the insult. The most interesting and widely studied TCS pathways will be discussed in the following paragraphs. Figure 2 shows an overview of the discussed ESR pathways in GNB. Interested readers for further reading on different TCSs can refer to these excellent articles [97,98,99,100].

#### 2.3.1. Regulator of Capsule Synthesis (Rcs) System

The Rcs (regulator of capsule synthesis) system is conserved in various members of the Enterobacteriaceae family, serving a major role in survival and virulence in some pathogenic species [101,102,103]. This non-orthodox TCS mainly responds to defects in LPS biosynthesis and transport, PG synthesis, and lipoprotein mislocalization by triggering the expression of genes involved in capsule synthesis, motility, biofilm formation, and virulence [103]. Studies have shown that the Rcs system governs the transition to specific physiological states, especially in the context of virulence, to adapt to the host environment [101]. The Rcs system has three main components (the so-called RcsCDB phosphorelay): RcsC (transmembrane hybrid kinase), RcsD (phosphotransfer transmembrane protein), and RcsB (response regulator). In the absence of any signal, the phosphatase activity of RcsC and RcsD maintain low levels of phosphorylated RcsB (RcsB-P), the active form of RcsB. The transcriptional regulator RcsB works as a homodimer or heterodimer with RcsA (in some cases, other ancillary proteins playing the role of RcsA are also involved), binding to different promoters. IgaA, an IM protein, functions as an inhibitor of the Rcs system which ensures that the system is not activated in the absence of environmental stimuli [104]. Although not exclusively, in most cases, Rcs activation involves the OM lipoprotein RcsF. When a stress perturbs the envelope or RcsF accumulates in the IM because of OM biogenesis defects, RcsF itself interacts with IgaA relieving the inhibition. It has been recently reported that in the OM, RcsF is complexed with the OMPs by BamA, and this association partially exposes RcsF to the cell surface, where it can monitor the integrity of the LPS layer [105] (Figure 2). Currently, there are two models to explain the activation of the phosphorelay by RcsF; according to the first model, in the absence of a stress envelope signal, BamA funnels RcsF through OmpA on the cell surface, preventing its interaction with IgaA. During envelope stress, BamA fails to bind RcsF which then interacts with IgaA and activates the Rcs system [104]. The alternative model postulates that RcsF monitors the state of LPS lateral interactions through its positively charged surface-exposed N-terminal domain. According to this model, when the LPS lateral interactions are disturbed, the state is sensed by RcsF and the signal is transduced through the C-terminal domain of RcsF to activate the Rcs system [106,107]. RcsF-independent signals are also present—for example, the deletion of *dsbA* (encoding for a periplasmic oxidoreductase protein required for flagellar assembly) can activate the Rcs system in the absence of RcsF in *Salmonella enterica* [108].

Rcs induction by environmental or by intrinsic stresses (mainly mutations) has the final purpose of reprogramming the transcriptome to make the cell more adapted to the changed environment or to improve a mutant membrane structure. The first study of Rcs activation was reported in response to osmotic upshift [104]. The initial response to Rcs activation in this case was the transient expression of the *cps* operon, required to produce colanic acid, an anionic polysaccharide that reinforces the cell envelope. Other known stimuli that activate Rcs system are lysozyme, which triggers the expression of lysozyme inhibitors, and mecillinam, which targets PG and induce the Rcs system [101,109]. Similarly, the oxidative damage to the OM in *S. enterica* serovar Typhimurium induces the Rcs system which in turn leads to the activation of the *dps* gene (encoding for the most abundant protein during the stationary growth phase that provides protection against reactive oxygen species that are produced by the host during infection), which relieves oxidative stress conditions, thus providing protection to the pathogen. Additionally, the Rcs system is implicated in OM modification in response to the membrane damage induced by cationic antimicrobial peptides (CAMPs) such as polymyxin B in *S. enterica* [110]. Another important cue for the activation of the Rcs system is the inhibition of PG synthesis by interaction of β-lactam antibiotics with the penicillin binding proteins (PMBs) [111]. Among the intrinsic sources of Rcs system activation, a few that are worth mentioning are envelope stress, such as *waaF* deletion, that leads to LPS core sugar deficiency [112], *lolA* mutations, which result in lipoprotein mislocalization [113], *tolA* deletion, leading OM perturbations [114], and PG modifications caused by deletion of *pbp4*, *pbp5*, *pbp7*, or *ampH*, to name a few [115]. Interestingly, *ugd* deletion (leading to the formation of a truncated LPS core in *E. coli*) also triggers activation of the Rcs system [116]. It has been shown that RcsF overexpression can activate the Rcs system; however, it is not proven that the normal induction signal works through increasing the level of RcsF for Rcs activation [117].

Due to its importance in the adaptation of the cell envelope to harsh environments, the Rcs system is involved in the virulence, intracellular survival, and persistence of many pathogenic species. For example, in *S. enterica* serovar Typhimurium, a *rcsC* mutant allele, encoding a protein with constitutive kinase activity, reduced the virulence of *S. enterica* in mice [118,119]. Also, a constitutively active *rcsC* has shown reduced phagocytosis rates of *Salmonella* cells by murine macrophages, which could be due to increased production of colanic acid [118]. The *rcsC* gene has also been found to be important for systemic infections in mice [120]. Interestingly, the *rcsC* mutation in *S. enterica* serovar Typhimurium has been found to affect the expression of *ugd* that is required for the synthesis and incorporation of L-aminoarabinose into LPS to induce bacterial resistance to polymyxin B [116]. The Rcs system has also been implicated in the temporal regulation of virulence gene expression during *S. enterica* serovar Typhimurium infection [121]. Similarly, in pathogenic enterohemorrhagic *E. coli* (EHEC) O157:H7, the Rcs system contributed to the adhesion and invasion of bacteria to the host cells [122]. The Rcs system is predominantly linked to controlling motility through inhibiting the expression of the *flhDC* operon, which encodes the master regulator of flagella production [123]. The role of the Rcs system in controlling flagellar motility through *flhDC* expression is conserved in species such as *S. enterica*, *Erwina amylovora*, and *Proteus mirabilis* [101], where it is known to affect cell motility, bacterial colonization, and biofilm formation. In *E. coli*, the activation of the Rcs system leads to increased transcription of the sRNA RprA, thereby negatively regulating biofilm formation by increasing the stability of the *rpoS* mRNA (encoding the stationary phase σ factor σ^S^) [124,125]. Furthermore, it has been shown that the Rcs system positively regulates the activity of PhoP/PhoQ TCS (discussed in Section 2.3.5), which in turn regulates *pagP*, a gene responsible for the modification of the LPS structure and implicated in the resistance of *Yersinia enterocolitica* to polymyxin B [126].

#### 2.3.2. CpxRA Two-Component System in Mediating Multidrug Resistance

CpxRA (from conjugative pilus expression) is a conserved TCS present in many species of *Enterobacteriaceae*. As a canonical TCS, this system consists of the sensor histidine kinase CpxA, its cognate response regulator CpxR, and CpxP, a periplasmic protein acting as negative regulator of the TCS [104]. CpxA senses specific signals and activates CpxR via phosphorylation. The phosphorylated CpxR regulates the expression of target genes by acting as a transcription factor. CpxP is a chaperone that directly interacts with misfolded proteins and binds CpxA to inhibit the system. The inhibition of the CpxRA is relieved by DegP, a periplasmic protease that degrades CpxP bound to the misfolded proteins, thus relieving the signal. NlpE, an OM lipoprotein, functions as the signaling module for the Cpx system and its overexpression activates CpxRA. The Cpx system responds to various signals including misfolded IM and periplasmic proteins, extracellular signals such as increased pH, altered membrane phospholipid composition, changes in lipoprotein production, overexpression of misfolded pilus subunits, high osmolarity stress, and PG perturbations [127] (Figure 2). Additionally, the Cpx system is activated by stresses affecting the bacterial envelope and plays a multifaceted role in GNB physiology, particularly in antibiotic resistance against various classes of antibiotics (including aminoglycosides, β-lactams, fosfomycin, and antimicrobial peptides [104]). Genetic analyses have shown that in *Salmonella* the Cpx system upregulates the expression of *amiA* and *amiC*, two N-acetylmuramoyl-L-alanine amidases, upon NlpE overexpression, thereby causing resistance to antimicrobial peptides, such as protamine, and α-helical peptides, magainin 2, and melittin [128]. The involvement of the Cpx system in protamine resistance was suggested by the observation that NlpE overexpression elevated the resistance only in the wildtype and not in the Δ*cpxR* mutant [128]. Among the genes regulated by the Cpx system in *E. coli*, *yqjA* is worth mentioning [129]*. yqjA* encodes for an IM transporter belonging to the DedA superfamily of membrane proteins and its deletion has been associated with increased sensitivity to alkaline pH, several antibiotics, and cationic peptides, as well as with cell division defects [130]. Interestingly, members of the DedA superfamily of transporters have recently been identified as lipid flippases involved in the final step of PG biogenesis, thus explaining the connection between these proteins and the resistance to the CAMP colistin in several Gram-negative species [131,132]. Another genome-wide susceptibility assay analysis in *E. coli* revealed that the CpxRA system is involved in the regulation of *tolC*, a gene encoding the OM component of multiple tripartite multidrug transporters implicated in providing high resistance against protamine [128]. The activation of the Cpx system leads to transcription of the *mar* locus, encoding for drug efflux pumps, which contributes to protamine resistance and possibly resistance against other CAMPs [128]. The role of *cpxA* in the context of antibiotic resistance has been shown by a study that explored the significance of a single-amino-acid substitution (Y144N) in the periplasmic sensor domain of CpxA. CpxA^Y144N^ conferred resistance in *E. coli* against β-lactams and aminoglycosides in a CpxR-dependent manner by regulating the expression of the OmpF porin and the AcrD efflux pump, respectively [133]. The Y144N substitution abrogates the interaction between CpxA and CpxP and increases phosphotransfer activity on CpxR, thereby activating the system. The addition of a strong AmpC (β-lactamase) inducer, such as imipenem, led to the abnormal accumulation of muropeptides in the periplasmic space, causing activation of the Cpx system. The suggested mechanism of activation is mainly through the interaction of muropeptide molecules with CpxP, which displaces CpxP from the sensor domain of CpxA [133]. The Cpx system is crucial for mediating antibiotic resistance also in *Salmonella enterica* serovar Typhimurium as *cpxR* mutants were found to be more susceptible to various antibiotics (including amikacin, gentamycin, apramycin, neomycin, ceftriaxone, ceftiofur, and cefquinone) compared to the multi-drug susceptible parental strain [93]. Another study observed that a CpxR overexpressing strain of *Salmonella* was more susceptible to colistin compared to the wild-type [134]. Additionally, the bactericidal effect of colistin in the CpxR overexpressing strain was potentiated with the exogeneous supplementation of citrate, α-ketoglutaric acid, and agmatine sulfate, suggesting CpxR, along with these metabolites, as adjuvants for colistin therapy [134]. Finally, the Cpx system has also been implicated in controlling fosfomycin resistance in enterohemorrhagic *E. coli* [135].

#### 2.3.3. BaeSR and AdeRS Two-Component Systems in Controlling Virulence

The BaeSR is a bacterial adaptive TCS present in many species of Enterobacteriaceae (Figure 2). The BaeSR system controls the expression of drug efflux pumps in *E. coli* and *A. baumannii* [136,137]. In *A. baumanni*, the BaeSR system regulates resistance to tigecycline through increasing the expression of the efflux pump genes *adeA* and *adeB* [138]. Additionally, the same study showed increased expression of drug efflux pumps *adeIKJ* and *macAB-tolC* after tigecycline induction [138]. The expression of the porin gene *ompW* in *S. typhimurium* was found to be affected by *baeR* deletion [139]. Another TCS, AdeRS is one of the best-characterized TCSs in *A. baumannii*, where it is associated with the global regulation of genes involved in sensing cell density/growth, or indirectly involved in sensing osmolality via the BaeSR system [140,141] (Figure 2). The AdeRS regulates the expression of efflux pump genes *adeABC*, coding for a member of the RND (Resistance–Nodulation–Division) family of efflux pumps implicated in resistance to aminoglycosides, erythromycin, tigecycline, chloramphenicol, trimethoprim, and fluoroquinolones [142]. Interestingly, the AdeABC pump has been associated with increased virulence, which could explain why many of the clinical isolates show mutations in the AdeRS system [143]. Several studies have highlighted the link between mutations in AdeR or AdeS and AdeABC efflux system-mediated multidrug resistance development, as the efflux pumps might be involved in the management of intracellular levels of the antibiotics [142,144,145]. The regulons of the BaeSR and AdeRS systems overlap, suggesting a crosstalk between these two systems [146,147].

Treating carbapenem-resistant *A. baumannii* infections is a serious problem in many countries. Cefiderocol, a novel siderophore-conjugated cephalosporin, presents an interesting treatment option with potent in vitro activity against *A. baumannii* [148]. A recent study explored the resistance mechanisms for cefiderocol in *A. baumannii* using sub-lethal doses of the antibiotic, which revealed mutations in the BaeS/R system associated with a 8- to 16-fold increased [149] cefiderocol minimal inhibitory concentrations (MIC), respectively [149]. Transcriptomic profiling of the mutants revealed upregulated expression of *macAB-tolC* and a major facilitator superfamily (MFS) efflux pump, suggesting a possible mechanism of resistance [149]. Moreover, the BaeR mutation resulted in increased virulence and biofilm formation in the mutant compared to the wild-type strain [149]. Similarly, a mutation in the BaeSR system has also been linked to cephalosporin resistance in *Salmonella enterica* serovar Typhimurium through overexpression of efflux pumps. In *Salmonella*, the BaeSR controls the expression levels of the outer membrane proteins STM1530 and OmpD, thus aiding in resistance against cephalosporin [150].

#### 2.3.4. Role of PmrAB Two-Component System in LPS Remodeling

PmrAB is the main TCS implicated in the modification of the LPS moiety in *E. coli*, *S. enterica*, *K. pneumoniae*, *Y. pestis*, *Citrobacter rodentium*, *Pseudomonas aeruginosa*, and *A. baumannii* [141]. In this system, PmrB acts as a histidine kinase and PmrA as its cognate response regulator (Figure 2). The most commonly observed remodeling mediated by *pmrAB* regulon are the modification of lipid A through palmitoylation, deacylation, or addition of 4-aminoarabinose (L-Ara4N) or phosphoethanolamine (pEtN). The addition of L-Ara4N is the most efficient way to reduce the net-negative charge of the membrane to zero, thereby leading to polymyxin resistance as the positively charged polymyxin fails to bind to the membrane. The modification mechanism appears to be conserved in other pathogenic species such as *K. pneumoniae*. In *P. aeruginosa*, the *pmrAB* regulates the expression of *cprA* gene which mediates polymyxin resistance and of other *pmr* genes (PA3552-PA3559) that regulate resistance against antimicrobial peptides [151,152]. Several studies have shown a link between mutations in the *pmrAB* operon and enhanced resistance to antimicrobial peptides which improves survival in chronic infections [141]. Accordingly, this TCS system has been involved in survival and persistence in chronic lung infections [153]. *A. baumannii* mutations in *pmrAB* have been shown to be associated with colistin resistance; however, the mechanism is unknown [154]. Notably, the acquisition of colistin resistance in *A. baumannii* was found to be associated with decreased virulence and fitness. However, these findings remain controversial as other studies did not find reduction in virulence and fitness in *A. baumannii pmrB* mutants and colistin resistance is generally associated with LPS loss [154,155].

One study showed that *pmrB* can also activate a non-cognate transcription factor, QseB, and this activation leads to enhanced tolerance against polymyxin B in uropathogenic *E. coli* strains [156]. QseB is the response regulator of another TCS, QseBC, that in pathogenic *E. coli* strains is implicated in biofilm formation and flagellar motility in response to quorum sensing [157]. A more recent study showed that QseB is directly involved in LPS modification even in the absence of the canonical LPS transcriptional regulator *pmrA*. In this study, QseB was found to control glutamate and 2-oxoglutarate metabolism, required for modification of lipid A in response to antibiotic exposure. [158]. This work nicely shows how metabolic rewiring controlled by QseB is responsible for stable resistance to positively charged antibiotics.

#### 2.3.5. PhoPQ Two-Component System

PhoPQ is another important TCS present in many bacterial species that is involved in sensing environmental perturbations, especially osmotic changes, magnesium depletion, and exposure to cationic antimicrobial peptides [159]. The sensor kinase PhoQ phosphorylates the regulator PhoP that controls the expression of many genes involved in survival and virulence [159] (Figure 2). The major downstream pathways controlled by PhoP induce LPS modification in response to CAMPs, low magnesium concentrations, and other physiological processes involved in envelope homeostasis, osmotic stress response, and redox balance [159]. A recent study showed the role of PhoPQ in acquisition of resistance to quinolones and cephalosporins in *Salmonella enteritidis* [160]. The *phoP* mutant, in response to nalidixic acid and ceftazidime exposure, showed a compromised membrane profile with respect to integrity, fluidity, and permeability [160]. PhoPQ was recently implicated in the development of resistance to last-resort antibiotics tetracycline and glycylcycline in *E. coli* through regulation of LPS modifications [161]. The resistant phenotypes were found to be caused by enhanced expression of phosphoethanolamine transferase EptB, which catalyzes the transfer of phosphoethanolamine to the lipid A core of the LPS [161]. Interestingly, the resistant phenotype was specific for EptB only and no other phosphoethanolamine transferases implicated in LPS modification were associated with resistance [161].

The PhoPQ system also plays an important role in regulating bacterial physiology in response to environmental stresses. The deletion of *phoPQ* genes in *Cronobacter sakazakii* negatively affects its biofilm formation [162]. For *Stenotrophomonas maltophilia*, the PhoPQ system has the most comprehensive response as it has been implicated in controlling swimming motility, antibiotic susceptibility, adaptation to stress, and virulence in nematodes [163].

### 2.4. Targeting TCSs for the Development of Novel Antimicrobial Drugs

As reported in the previous paragraphs, the ESR systems play a crucial role in the adaptation of GNB to harsh conditions, including the host environment, and in maintaining cell envelope homeostasis. Therefore, it is not surprising that research on these systems, especially on TCSs, is gaining importance in the context of the urgent need for the development of novel antimicrobial strategies. The first synthetic inhibitor of a bacterial TCS, a molecule targeting the *P. aeruginosa* AlgR1–AlgR2 signaling system involved in synthesis of the major exopolysaccharide for biofilm formation (alginate), was reported in 1993 [164]. While not properly directed against an ESR, the mechanism of action of this inhibitor showed for the first time that TCSs can be targeted for the design of innovative antibacterial molecules. Since then, significant research has been conducted to identify more TCS inhibitors and several examples have been described, a few of them targeting TCSs involved in envelope homeostasis (the list of a few known TCS inhibitors is given in Table 2). Interested readers can refer to the following articles for further reading on the topic [97,98,165,166,167,168].

At present, there are several reports that describe natural and synthetic compounds targeting TCSs, showing antimicrobial activity against pathogenic bacteria; however, more research is needed in the area to gain an understanding of the specific mechanisms of action of these drugs.

Some of the TCSs directly modulate essential processes in bacteria such as growth and survival, which makes them a good target for the development of bactericidal antibiotics. For example, targeting the ArsS/ArsR system, which is essential for the growth of *Helicobacter pylori*, can be a good bactericidal strategy in the acidic stomach environment [176]. Other examples of inhibitors are some that target ATP-binding domains like radicicol, a putative ATP competitor which can bind to the unique ATP catalytic domain (called the Bergerat fold) present in many sensor kinases including PhoQ [174]. Thus, the identification of inhibitors targeting this fold can recognize multiple sensor kinases with the same domain. Radicicol has been shown to inhibit the autokinase activity of PhoQ which prevents phosphorylation-based activation of PhoP regulator, consequently affecting expression of virulence-specific genes in *Salmonella* and reducing its survival within macrophages [174]. As shown earlier, PhoP is implicated in the regulation of genes responsible for acquisition of antimicrobial resistance such as *pmrA* in *A. baumannii*, *P. aeruginosa*, and *E. coli*. CAMPs, such as polymyxin B, are employed as a last-resort antibiotic for the treatment of infections caused by GNB that are resistant to commonly used antibiotics, such as quinolones, aminoglycosides, and ß-lactams. Radicicol and the inhibition of PhoQ could function as antibiotic adjuvant for colistin therapy in MDR infections. PhoPQ is also involved in virulence in pathogenic species like *Shigella*; therefore, targeting this system can be useful for treating infections caused by these bacteria. The inhibitors discussed here (Table 2) were identified through a virtual screening method based on the ATP catalytic domain of PhoQ from *Shigella flexneri* which inhibit kinase activity resulting in compromised invasion of HeLa cells by *S. flexneri* [171]. Interestingly, despite the conservation of the target PhoQ fold, no apparent cytotoxic effects and hemolytic activities were observed with these inhibitors, suggesting their safe nature for use as pharmaceutical drugs.

Small-molecule inhibitors such as LED209 have also shown promise in preventing the virulence behavior of pathogenic bacteria. LED209 targets QseC sensor kinase, which regulates several virulence genes and functions as a prodrug, releasing the active compound OM188, which inhibits QseC activity and reduces virulence in bacteria like *S. typhimurium* and *Francisella tularensis*. It should be noted that the QseBC TCS is highly conserved in many of the Gram-negative pathogens, including enterohaemorrhagic *E. coli* (EHEC), uropathogenic *E. coli*, and *Salmonella enterica*, suggesting a possible common mode of mechanism for virulence and pathogenesis. In EHEC, the inhibitor LED209 affects pathogenesis by decreasing the QseB-induced expression of LEE pathogenicity island genes [170]. Maprotiline, an FDA-approved antidepressant, was identified in a screening as a QseC inhibitor which displayed antibiofilm activity against the human pathogen *F. novicida* through a predicted interaction with the periplasmic sensor domain of the histidine kinase, QseC [169]. Maprotiline exerts its antibiofilm effects through downregulating the expression of the virulence factor *iglC*, a *Francisella* Pathogenicity Island (FPI) gene. Interestingly, maprotiline also showed a promising profile in an *F. novicida* infection mice model in terms of survival and disease onset. The current data validate these TCS inhibitors as promising drug targets, in particular as antibiotic potentiators to treat antimicrobial resistant GNB infections.

## 3. Limitations Associated with the Development of ESR Inhibitors

Despite the promising potential of targeting ESRs for the development of new antibacterials or adjuvants for anti-biotic therapy, there are still many limitations preventing the identification of clinically effective molecules. The first obstacle for a potential ESR (especially TCS) inhibitor is common to any other antibiotic—the need to cross the OM permeability barrier to reach the sensory element of the system. This issue could potentially be managed with the use of “Trojan horse” compounds, which escort the molecule into the periplasm. One such example is the use of the iron-binding moieties of siderophores to ESR inhibitors to allow access through iron-uptake systems [177]. Fortunately, the ESR components and their regulatory members are located in the IM and can be easily targeted using the “Trojan horse” strategy. Together with design improvements using the concepts of medicinal chemistry to adapt the existing ESR inhibitors, a combinatorial approach exploiting the synergistic effect of other antibiotics could be adopted. It should be noted that the use of “Trojan horse” compounds, while potentially a successful strategy, may be susceptible to pre-existing resistance mechanisms in the target bacterium.

Another limitation of ESR inhibitors is their efficacy, which may vary based on the host’s physiological state. For example, inhibitors targeting TCSs may be rendered ineffective due to high-affinity interactions between HKs and their corresponding response regulators, which is difficult to disrupt. Additionally, there is a risk involved in targeting envelope stress responses as it may inadvertently lead to system hyperactivation, leading to increased antimicrobial resistance. Moreover, while ESR inhibitors should have no direct impact on cellular survival and are not intended to suppress bacterial growth or cause cell death, their impact on the host microbiome is difficult to predict because ESR homologs can be found across a wide range of microbiome taxa.

HKs are promising targets for the development of ESR inhibitors, and numerous studies have found inhibitors targeting HKs in a variety of bacteria (as discussed in Section 2.4); nevertheless, substantial challenges with their screening and development persist. For example, although HKs are not present in mammals, some mammalian proteins, including MutL mismatch repair protein, Hsp90 chaperone, and certain mitochondrial kinases, contain the unique Bergerat ATP binding structure found in bacterial HKs. Accordingly, the previously mentioned radicicol also recognizes mammalian α-keto acid dehydrogenase, which harbors the Bergerat fold. This commonality makes it difficult to create inhibitors that selectively target bacterial HKs while avoiding other important proteins of the mammalian host containing an analogous structural motif [178]. On the other hand, because bacterial HKs regulate a wide range of physiological processes, inhibitors targeting HKs can be designed to serve multiple purposes, including antibiotic potentiators, biofilm inhibitors, and bacteriostatic/bactericidal agents, depending on the physiological activities of the targeted HKs in bacteria. Due to high structural similarities between various HKs, an inhibitor of one HK theoretically has the potential to block multiple TCS regulatory networks, being beneficial for inhibitor development. However, inhibiting multiple essential HKs might exert selective pressure on bacteria, especially if the inhibitor targets essential HKs, inevitably leading to the emergence of resistance. Understanding how bacteria develop resistance to HK inhibitors in a clinical setting is crucial in developing an effective antimicrobial therapy targeting HK inhibitors [98].

Another challenge in the development of these inhibitors is the choice of suitable screening assays. Since inhibitors targeting non-essential HKs possess minimal or no classical antibacterial effects, simple routinely used in vitro assays, such as MIC determination, cannot be utilized for the confirmation of the inhibitors’ activity. Furthermore, while inhibitors with promising MIC values (in the low μg/mL concentration range) are suitable for clinical application, their performance in in vivo systems is questioned. The drug screens should be conducted in conditions that mimic the host environment. For example, using models such as *Caenorhabditis elegans*, tissue models such as macrophages, human blood as a growth medium, and simulated bodily fluids can help identifying compounds with greater in vivo efficacy and relevance while ruling out compounds with higher toxicity [90].

It is worth mentioning that, in general, it is difficult to determine whether the antibacterial action observed in vivo is a direct result of HK inhibition or whether the bactericidal impact is unrelated to HK activity [168]. The development of phosphohistidine-specific antibodies could facilitate the establishment of assays to directly assess the inhibition of HKs in vivo, allowing researchers to confirm whether in vitro activity translates to actual target engagement in living organisms.

Besides the need for effective in vitro and in vivo assays to evaluate the activity of HK inhibitors, another limitation in designing HK inhibitors is the lack of understanding of the interactions between the targeted HK domain and the potential ligands, which is required to improve target specificity and antibacterial activity. In any case, following the identification of a potential inhibitor, the next steps should be antibacterial activity tests in vivo, toxicity trials, and preclinical research before the ESR inhibitors are introduced into clinical settings. Finally, most of the work in the direction of development of new antibiotics, including HK inhibitors, has primarily been driven by academic laboratories with limited resources. The absence of support from the pharmaceutical sector impedes the advancement of promising substances through clinical trials and further development.

## 4. Conclusions and Future Directions

TCS inhibitors are ideal candidates for developing novel antimicrobial drugs, particularly for combating drug-resistant GNB infections. Despite this, to date, only a few inhibitors have been reported. As TCS inhibitors act by disrupting upstream regulatory mechanisms, they could be effective against MDR pathogens that have developed resistance to traditional antibiotics. Moreover, many of the antimicrobial resistance genes are under the direct or indirect control of TCSs; therefore, inhibitors of these systems could work as antibiotic adjuvants to potentiate the efficacy of the existing antibiotics. Therefore, TCS inhibitors could be used in combination with conventional antibiotics to enhance their effectiveness. For example, some inhibitors can re-sensitize resistant bacteria to antibiotics by disrupting biofilm formation.

Moreover, targeting non-essential TCS involved in virulence or antibiotic resistance presents an opportunity to design lead drugs with a lower potential for resistance development as these inhibitors would exert less selective pressure on bacterial survival, that potentially increase the probability to develop resistance. However, challenges remain for the design and screening of these TCS inhibitors. A major challenge is developing highly specific inhibitors that effectively target bacterial TCSs without interfering with human cell signaling pathways. Addressing potential toxicity concerns and ensuring the safety of these inhibitors is therefore crucial. Furthermore, research should be directed at understanding the possible mechanisms by which bacteria can acquire resistance against these inhibitors, in order to develop counterstrategies. Many TCS inhibitors exhibit promising results in in vitro studies. However, their effectiveness and safety need to be rigorously tested in animal models and eventually in human clinical trials.

In conclusion, TCS inhibitor research is a fairly novel area, and continued efforts are needed to identify and validate novel TCS targets and to develop new classes of inhibitors with improved potency, specificity, and pharmacological properties. Targeting TCSs involved in regulating virulence factors like toxins, adhesins, and biofilm formation could lead to new therapeutic options, especially for chronic infections where reducing bacterial virulence might be sufficient to control the infection. Given the high degree of structural similarity between certain bacterial HKs, researchers are exploring the possibility of developing inhibitors that can simultaneously target multiple TCSs. Importantly, it should be considered that targeting bacterial histidine kinase-based sensor proteins allow minimum risk of host toxicity, as the eukaryotic signal transduction systems are serine/threonine-based sensor kinases unlike the bacterial histidine kinases [98]. This “polypharmacolgical” approach could enhance antibacterial activity and potentially slow down the development of resistance. Also, research should be directed on understanding the complex interplay between different TCSs within a bacterium and how this crosstalk might contribute to antibiotic resistance. Disrupting these signaling networks could offer new ways to combat resistance. Continued research focused on addressing the challenges of target specificity resistance and in vivo validation will be essential for translating the promise of TCS inhibitors into effective therapies.

## Figures and Tables

**Table 1:** List of OM biogenesis inhibitors and their mode of action in Gram-negative bacteria.

OM Biogenesis Inhibitors in Gram-Negative Bacteria					
S.I. No.	Inhibitor Compound	Target	Bacterial Species	Mechanism of Action	Source
1	Fmoc-β-(2-quinolyl)-d-alanine	SurA	*E. coli*	Binding to SurA client site to inhibit chaperoning activity	[71]
2	MAB1	BamA	*E. coli*	Directly binds to extracellular epitope and inhibits β-barrel folding activity	[66]
3	MRL-494	BamA	*E. coli*	Inhibition of OMP biogenesis	[67]
4	Darobactin	BamA	*Multiple GNB*	Binds and selectively induces the closed-gate conformation of BamA-β	[68]
5	JB-95	BamA, LptD	*P. aeruginosa*, *A. baumannii*, *E. coli*	Disruption of the OM through interaction with β-barrel proteins	[87]
5	Compound **2** (pyrazole)	LolCDE	*E. coli*	Inhibition of lipoprotein trafficking to the OM, toxic mislocalization of Lpp	[83]
6	Compound **1**	LolCDE	*E. coli*	Inhibition of lipoprotein trafficking to the OM, toxic mislocalization of Lpp	[82]
7	G0507	LolCDE	*E. coli*	Inhibition of lipoprotein trafficking to the OM, toxic mislocalization of Lpp	[81]
8	G907	MsbA	*E. coli*	Traps MsbA conformation inward facing, blocking translocation of LPS	[73]
9	Compound **1**	MsbA	*A. baumannii*	Stimulates ATPase activity whilst decoupling it from LPS translocation	[78]
10	Thanatin	LptAC	*E. coli*	Blocks the interaction between LptA subunits and between LptA/C	[74,75]
11	Zosurabalpin	LptBFGC	*A. baumannii*	Binds LptF, LptG, and LPS within the substrate cavity, stalling the Lpt system	[76,77]
12	POL7001/POL7080	LptD	*P. aeruginosa*	Disruption of LPS flow through LptD	[79]

**Table 2:** List of known TCS inhibitors and their mode of action in Gram-negative bacteria.

Known TCS Inhibitors in Gram-Negative Bacteria					
S.I. No.	Inhibitor Compound	Target	Bacterial Species	Mechanism of Action	Source
1	Maprotiline	QseC	*Francisella novicida*	Interacts with the periplasmic sensor domain of QseC, reducing biofilm formation	[169]
2	LED209	QseC	*S. typhimurium* and *F. tularensis*	Inhibits QseC ligand binding and the resulting autophosphorylation without impacting bacterial viability but critically disabling several virulence mechanisms; demonstrated efficacy in a mouse infection model	[170]
3	Cai compounds (Cai-**1**, Cai-**2**, Cai-**3**, and Cai-**4**)	PhoQ	*Shigella flexneri*	Inhibits autophosphorylation activity of PhoQ	[171]
4	Velikova-13 *	PhoR	*E. coli*	Inhibits the autophosphorylation of HK in a concentration-dependent manner	[172]
5	NSC48630	PhoP	*Salmonella enterica*	Inhibits the formation of the *S. enterica* PhoP–DNA complex	[173]
6	Radicicol (Eukaryotic Hsp90 inhibitor)	PhoQ	*E. coli*	Interacts with the ATP-binding pocket of bacterial sensor kinase PhoQ	[174]
7	2-aminobenzothiazole compounds (**21** and **33**)	PhoQ	*S. enterica*	Inhibits PhoQ and affect bacterial growth and resistance to polymyxin B and E	[175]

* Predominant activity against Gram-positive bacteria.
